# Comparison of pharmacodynamics and pharmacokinetics of ultra‐rapid‐acting insulin aspart and rapid‐acting insulin aspart around continuous moderate intensity exercise in adults with type 1 diabetes: A randomised controlled trial

**DOI:** 10.1111/dom.70487

**Published:** 2026-02-10

**Authors:** Jason P. Pitt, Alexander Müller, Chloe Nicholas, Olivia M. McCarthy, Othmar Moser, Stephen C. Bain, Harald Sourij, Richard M. Bracken

**Affiliations:** ^1^ Applied Sport, Technology, Exercise and Medicine Research Centre Swansea University Swansea UK; ^2^ Cardiometabolic Trials Unit Medical University of Graz Graz Austria; ^3^ Steno Diabetes Center Copenhagen University Hospital Copenhagen Denmark; ^4^ Department of Human Movement Science Sport & Health, Exercise Physiology, Training & Training Therapy Research Group, University of Graz Graz Austria; ^5^ Division of Endocrinology and Diabetology Medical University of Graz Graz Austria; ^6^ Swansea University Medical School Swansea University Swansea UK; ^7^ Health Technology Solutions, Interdisciplinary Research Institute Swansea University Swansea UK

**Keywords:** exercise, insulin analogues, pharmacodynamics, pharmacokinetics, type 1 diabetes

## Abstract

**Aims:**

To compare the effects of dose reductions of ultra‐rapid‐acting insulin aspart (URA‐IAsp) and rapid‐acting insulin aspart (IAsp) on blood glucose concentrations during continuous moderate‐intensity exercise in people with type 1 diabetes (T1D).

**Materials and Methods:**

In this double‐blind, laboratory‐controlled study, 43 adults with T1D completed four experimental visits in a randomised crossover design. Participants injected a 50% or 75% reduced dose of URA‐IAsp or IAsp with a standardised breakfast 60 min prior to 45 min of cycling at ~61% V̇O_2peak_. The same insulin type and dose were administered 4 h after the first injection, alongside an identical lunch meal. Venous blood samples were taken at 5‐, 10‐, and 15‐min epochs, for a total of 70 timepoints, throughout the trial day until 4 h after the second injection to determine blood glucose and insulin concentrations. The primary endpoint was the four‐way comparison of blood glucose change from exercise start to end.

**Results:**

Blood glucose declined during exercise to a similar extent between 50% dose URA‐IAsp (−4.0 ± 2.8 mmol L^−1^) and all other conditions (all *p* > 0.05), yet fell more in the 50% IAsp dose (−5.1 ± 3.0 mmol L^−1^) compared to the URA‐IAsp (−2.8 ± 3.3 mmol L^−1^) and IAsp (−3.4 ± 3.3 mmol L^−1^) 75% reduced dose conditions (both *p* < 0.05). Differences in blood insulin concentrations between trials were only resultant of insulin doses and not insulin type from 30 min after the first insulin injection.

**Conclusions:**

Insulin dose reductions around acute moderate‐intensity exercise yield similar glucose‐lowering effects with URA‐IAsp and IAsp. The extent of dose reductions exerts greater influence on glycaemia than the type of fast‐acting insulin.

## 1 INTRODUCTION

The latest generation of commercially available ultra‐rapid‐acting insulins — faster‐acting insulin aspart (URA‐IAsp, Fiasp®) and ultra‐rapid lispro insulin (Lyumjev®) – present faster pharmacodynamic and pharmacokinetic (PD/PK) profiles. Compared to their predecessors (insulin aspart [IAsp, NovoRapid®/NovoLog®] and insulin lispro [Humalog®], respectively), ultra‐rapid‐acting insulins demonstrate a left‐shift in PD/PK characteristics, with earlier onset and a greater early insulin effect.^1^, ^2^, ^3^ These properties allow ultra‐rapid‐acting insulins to be administered closer to the anticipated postprandial glucose peak (e.g., immediately before eating).

URA‐IAsp was the first ultra‐rapid‐acting insulin to be approved by the FDA. A series of studies, the ‘ONSET’ trials, were conducted to establish PD/PK comparisons between URA‐IAsp and IAsp and to compare long‐term glucose control outcomes across different populations. In adults with type 1 diabetes (ONSET 1), URA‐IAsp was shown to provide favourable post‐prandial glucose control and similar or favourable HbA_1c_ over 26 and 52 weeks (both ONSET 1 trials)^4^, ^5^ and when participants were using either detemir (ONSET 1) and degludec (ONSET 8)^6^ basal insulins.

To mitigate the risk of hypoglycaemia during exercise, international guidelines recommend reducing the bolus insulin dose administered with a meal prior to engaging in continuous moderate‐intensity exercise.^7^, ^8^ It is plausible that transitioning from rapid‐acting to ultra‐rapid‐acting insulin may lead to different glucose outcomes under the same exercise protocol and the same pre‐exercise mealtime insulin administration protocol, particularly when exercise occurs within the primary window of insulin action (~2 h).

Literature investigating the use of the latest generation of mealtime insulins around exercise is limited. Molveau et al.^9^ have previously compared the use of URA‐IAsp to IAsp under different timing conditions prior to exercise when using 50% dose reductions only. It was found that the decline in blood glucose during exercise was less pronounced when administering URA‐IAsp instead of IAsp, independent of whether the dose was administered alongside a mixed meal either 60 or 120 min prior to moderate‐intensity cycling exercise. Nevertheless, there are no data currently available to investigate different dose reductions of an ultra‐rapid‐acting insulin around exercise, with a comparator rapid‐acting insulin. As ultra‐rapid‐acting insulins become increasingly integrated into clinical practice, timely data on their comparative effects on blood glucose during exercise would be valuable for refining insulin reduction recommendations.

The aim of this study was to compare the effect of an URA‐IAsp and a rapid‐acting insulin (IAsp) on blood glucose concentrations in response to equivalent peri‐exercise dose reductions. The primary outcome assessed the effects of dose reduction of insulin type on the change of blood glucose during exercise.

## 2 MATERIALS AND METHODS

### 2.1 Study design

This study was a prospective, two‐site, double‐blind, randomised four‐arm cross‐over clinical trial (German Clinical Trials Register DRKS00015855; Eudra‐CT 2019–001281‐14). The study was performed in accordance with the Declaration of Helsinki (1996) and Good Clinical Practice. Ethical approval was provided by national ethics committees (18/WA/0421 and EK 31–314 ex 18/19).

### 2.2 Screening visit

Participants were invited to attend a screening visit to take informed consent, gauge eligibility for the study, take baseline measurements, and to perform a cardiopulmonary exercise test (CPET). Key eligibility criteria included: 18–65 years of age (inclusive), T1D ≥12 months, multiple daily injection regimen ≥12 months, and HbA_1c_ ≤ 9.5% [80.3 mmol.mol^−1^]. After trial inclusion, participants were switched to a basal‐bolus regimen of insulin degludec (Tresiba®, Novo Nordisk A/S, Denmark) and IAsp (Novorapid®, Nordisk A/S, Denmark), unless already prescribed. Patients already using URA‐IAsp [Fiasp®, Nordisk A/S, Denmark] were not switched to IAsp. Insulin therapy dosing was adapted where necessary to ensure stable glucose control.

### 2.3 Experimental trials visits: Trial protocol

This study consisted of four experimental trial visits, each representing one of the four trial arms, in a randomised crossover design. Participants arrived at the research facility on an experimental trial day at 07.30, with the prerequisites of being fasted, without having taken a bolus insulin dose for the preceding 5 hours, and without having undergone strenuous physical activity, consuming alcohol, or had blood glucose concentrations of <3.0 mmol L^−1^ for the previous 24 hours as assessed by continuous glucose monitor (CGM; FreeStyle Libre 2, Abbott, USA). Participants had a cannula inserted into a vein in the antecubital fossa and had an electrocardiogram (ECG) Holter monitor fitted (eMotion Faros 180°, Bittium Biosignals Ltd., Finland). At 08.00, basal insulin degludec was taken as per usual regimen (one participant took a split dose of insulin degludec the evening before and on the morning of each trial day). At 08.30, participants injected a reduced dose of mealtime insulin subcutaneously into the abdomen according to their assigned randomised trial arm: (1) 50% reduced dose of URA‐IAsp (U50), (2) 75% reduced dose of URA‐IAsp (U75), (3) 50% reduced dose of IAsp (A50), (4) 75% reduced dose of IAsp (A75). Both researchers and participants were blinded to insulin dose and type. Insulin doses for 50% and 75% reductions were calculated based on what the individual participant would normally administer for the meals and insulin pens were prepared for injection independently of the research team. Immediately after, participants consumed a drink meal (Fortijuce, Nutricia, Netherlands [Macronutrients per 100 mL: carbohydrates 33.5 g, fats 0.0 g, protein 3.9 g]) equating to 1 g carbohydrate per 1 kg of body mass. Participants were allowed no longer than 5 min from the point of insulin injection to consume the drink meal.

At 09.30, the exercise protocol commenced, consisting of a 3‐min warm‐up (20–40 W) and 42 min of moderate‐intensity exercise on a cycle ergometer (Lode Corival CPET, Cranlea, UK) cycling at 70–80 revolutions per minute. Experimental trial visits' (moderate) exercise intensity was calculated as the midpoint between lactate turnpoint 1 and lactate turnpoint 2 achieved during the CPET.^10^

An identical carbohydrate‐containing drink and (reduced) insulin dose from the morning protocol were taken at 12.30. Participants were rested throughout the entire trial day, except for the exercise session, until 16.30 when the trial concluded. Trial day protocol times, as listed above, were kept consistent and accurate between and within participants to promote robust data collection methods (Figure S1). Water was consumed ad libitum. Hypoglycaemia (≤3.9 mmol L^−1^) was treated with 10–20 g of high glycaemic index carbohydrates (Lift Glucose Shots, Lift, UK) once every 15 min until blood glucose ≥4.0 mmol L^−1^ (Table S1).

### 2.4 Experimental trials visits: Methods of testing

To track PD/PK changes, venous blood samples were drawn in pairs of two separate 1.2 mL samples throughout the trial day. One sample from the pair was dedicated to the analysis of plasma glucose (collected via 1.2 mL S‐Monovette Glucose, Sarstedt, Germany) and one sample was dedicated to the analysis of serum insulin concentrations (collected via 1.2 mL S‐Monovette Serum, Sarstedt, Germany). Time intervals in between venous sample timepoints were either 5, 10, or 15 min, equating to 70 scheduled pairs of venous blood samples per trial day per participant (Figure S1). Plasma and serum samples were frozen at −80°C immediately after centrifugation until analysed for glucose (analysed via Cobas® C501, Roche, Switzerland) and insulin concentrations (analysed via ADVIA Centaur® XPT, Siemens Healthineers, Germany), respectively.

CGM, spirometry (METAMAX 3B, Cortex, Germany), ECG, and heart rate (Polar T31, Polar, Finland) data were measured continuously throughout exercise. Finger‐prick capillary blood glucose (FreeStyle Libre, Abbott, USA), earlobe capillary blood glucose and lactate (Biosen C‐Line, EKF Diagnostics, Germany), and rating of perceived exertion (Borg Scale^11^) were taken immediately before the warm‐up, immediately after the warm‐up, once every 6 min during exercise, and immediately after exercise. Blood ketones (FreeStyle Libre, Abbott, USA) were measured when blood glucose was ≥17.0 mmol L^−1^.

### 2.5 Data analysis

All statistical analyses were performed on SPSS 29.0 (IBM, USA). The primary endpoint of this study was the comparison between the change in blood glucose from exercise start to exercise end between the four trial arms. As *n* < 50, normality testing was carried out using the Shapiro–Wilk test.^12^ Comparisons between the four trial arms were made using repeated‐measures analysis of variance (ANOVA). Sphericity testing was performed using Mauchley's test of sphericity. Where the assumption of sphericity was violated, Greenhouse–Geisser correction was implemented. In instances of ANOVA significance, post‐hoc analysis via Bonferroni‐corrected t‐tests was performed to identify significance between all possible group pairings.

Comparisons between screening visit and final visit data were made using the paired samples t‐test. Metrics relating to maximum concentration (C_max_) and time to maximum concentration (t_max_) were individualised by identifying each participant's C_max_ and t_max_. Incremental area under the curve (AUC) was calculated via trapezoidal method.^13^ To identify outliers in time series data, an adjusted Hampel filter^14^ was employed, as similarly used in recent analyses of blood glucose‐related time series data.^15^, ^16^ Adjustment was made after assessment of its aggression or leniency towards known outliers and known acceptable, but extreme, values (e.g., the apex or nadirs of steep curves). Where an outlier was identified, the value was checked by a researcher (e.g., via visual inspection of a box and whisker plot) before a decision on removal from the dataset.

## 3 RESULTS

### 3.1 Participant characteristics

Participant baseline characteristics determined at the screening visit are detailed in Table 1. There were no differences between body mass or HbA_1c_ metrics at the screening versus final visit (all *p* > 0.05). A total of 23 578 out of a possible 24 220 venous blood samples (97.3%) were taken in this study. 20 venous blood sample datapoints were excluded via filtration of outliers. Of 44 participants randomised to the study experimental visits, one dropout occurred after the participant's first experimental visit for personal reasons. Data for 44 participants were retained for intention to treat analysis; however, analyses relating to multi‐trial comparisons for the one dropout were unavailable.

**TABLE 1:** Participant characteristics at screen visit (*n* = 44 randomised).

Characteristic	Screen visit	
	Mean ± SD	Range
Participant information		
Sex (male:female)	30:14	
Age (years)	39 ± 13	19–62
Anthropometry		
Body mass (kg)	77.5 ± 13.8	58.5–126.9
BMI (kg m^−2^)	24.4 ± 3.5	19.2–38.3
Diabetes information		
Diabetes duration (years)	15 ± 12	1–49
HbA_1c_ (%)	6.9 ± 1.0	5.1–8.8
HbA_1c_ (mmol mol^−1^)	51.6 ± 10.5	32.2–72.7
Pre‐study total daily insulin dose (IU kg^−1^)	0.57 ± 0.27	0.24–1.51
Insulin dosage for 50% reduced conditions (IU)	4.9 ± 5.2	2–36
Insulin dosage for 75% reduced conditions (IU)	2.5 ± 2.6	1–18
Cardiopulmonary exercise test information		
V̇O_2peak_ (L min^−1^)	2.85 ± 0.80	1.78–5.12
V̇O_2peak_ (mL kg^−1^ min^−1^)	36.7 ± 9.0	20.0–58.0
Power_peak_ (W)	220 ± 59	133–380
Blood lactate_peak_ (mmol L^−1^)	9.3 ± 2.3	4.2–13.1
Heart rate_peak_ (beats min^−1^)	175 ± 14	153–202

Abbreviations: BMI, body mass index; HbA1c, glycated haemoglobin. V̇O_2peak_, peak volume of oxygen uptake.

### 3.2 Primary outcome: Blood glucose during exercise (60–105 min)

The (non‐relativised) immediate pre‐exercise blood glucose concentrations were similar across all conditions (U50 16.9 ± 4.0, U75 17.7 ± 4.0, A50 17.1 ± 4.1, A75 17.9 ± 4.3 mmol L^−1^; *p* = 0.263). Blood glucose declined from concentrations measured immediately before exercise to those immediately after exercise (U50–4.0 ± 2.8, U75–2.8 ± 3.3, A50–5.1 ± 3.0, A75–3.4 ± 3.3 mmol L^−1^; *p* < 0.001) with post‐hoc analysis revealing that the decline in blood glucose in U50 was not different to all other conditions (Table 2; all *p* > 0.05). A50 fell to a greater degree than both U75 (*p* < 0.001) and A75 (*p* = 0.001), which themselves fell similarly to each other (*p* = 1.000). There were no differences between conditions in any spirometry‐derived or exertion‐related exercise data (Table S2; all *p* > 0.05).

**TABLE 2:** Blood glucose concentrations during 45 minutes of moderate‐intensity cycling exercise (60–105 min).

Parameter	U50	U75	A50	A75	*p* value
Exercise start BG_R0_ (mmol L^−1^)	+7.1 ± 2.8^a^	+8.3 ± 2.2^b^	+7.0 ± 2.6^bc^	+8.6 ± 1.9^ac^	<0.001*
*C*_max0–105min_ (mmol L^−1^)	+8.4 ± 2.7^ab^	+9.9 ± 2.1^ac^	+8.3 ± 2.3^cd^	+10.0 ± 2.2^bd^	<0.001*
*t*_max *C*max0–105min_ (min)	65.8 ± 14.1^a^	73.6 ± 11.2^ab^	63.1 ± 11.4^bc^	72.1 ± 11.1^c^	<0.001*
Difference between baseline (0 min) and end of exercise BG (mmol L^−1^)	+3.0 ± 3.2^ab^	+5.6 ± 3.4^ac^	+2.0 ± 3.3^cd^	+5.0 ± 3.3^bd^	<0.001*
Reduction in BG from start to end of exercise (mmol L^−1^)	−4.0 ± 2.8	−2.8 ± 3.3^a^	−5.1 ± 3.0^ab^	−3.4 ± 3.3^b^	<0.001*
AUC_60–105min_ relative to exercise start (pmol min L^−1^)	−44 ± 71	−16 ± 82^a^	−68 ± 84^a^	−42 ± 91	0.014*
AUC_0–105min_ (mmol min L^−1^)	483 ± 194^a^	601 ± 142	509 ± 194^b^	604 ± 147^ab^	0.003*

*Note*: All data relativised to resting blood glucose concentration taken at baseline (BG_R0_) unless otherwise indicated. *C*_max0–105min_, individualised maximum blood glucose concentration between rest (0 min) and end of exercise (105 min); *t*_max *C*max0–105min_, individualised time until maximum blood glucose concentration between rest (0 min) and end of exercise (105 min); ^a,b,c,d^ represent statistically significant (*p* ≤ 0.05) post‐hoc comparison between two conditions.

Abbreviations: AUC, area under the curve (incremental); BG, blood glucose.

* Denotes statistical significance for main effect.

### 3.3 Secondary outcomes: Trial day blood glucose concentrations

**FIGURE 1:**
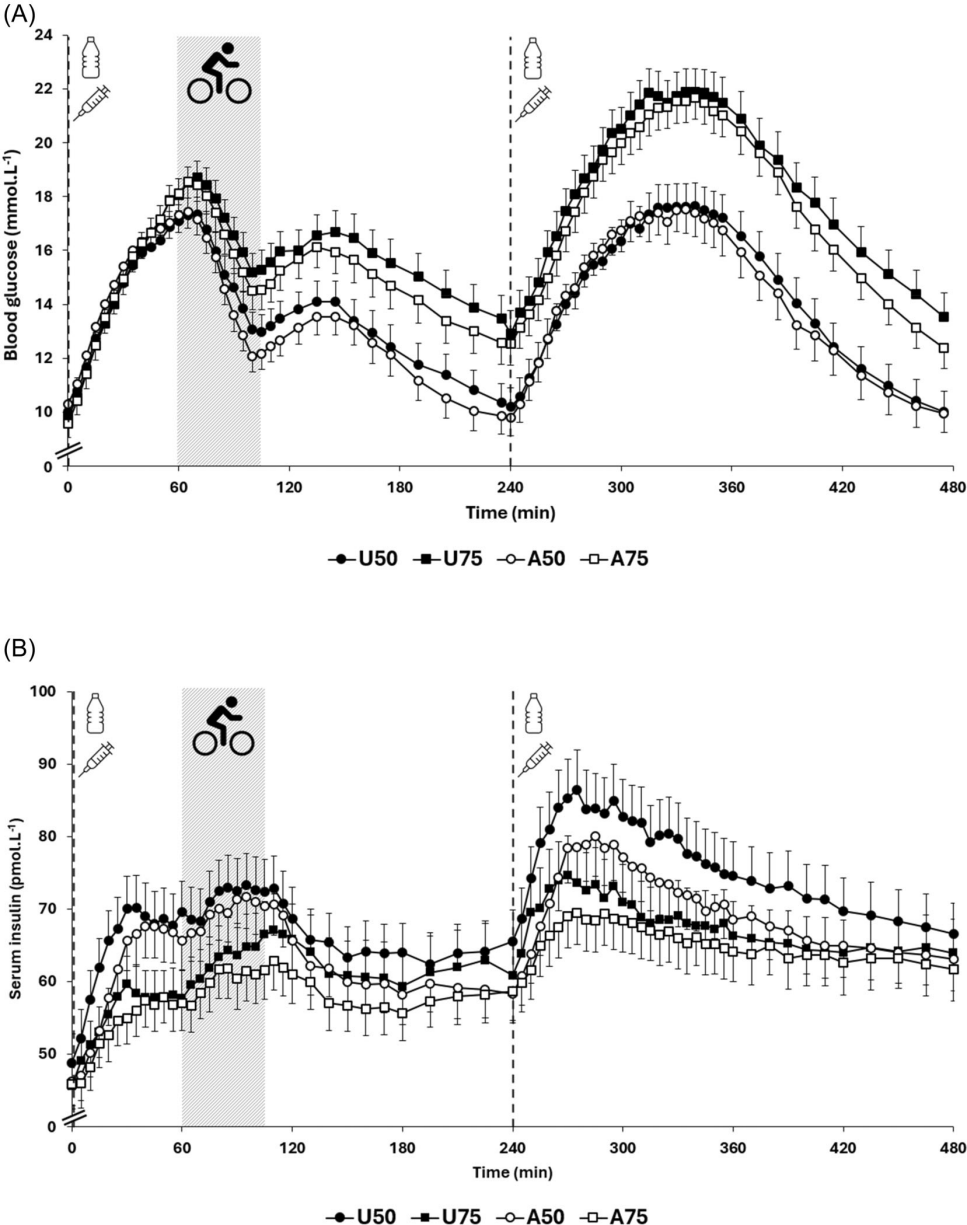
Blood glucose (A) and serum insulin concentrations (B) over the whole trial day (0–480 min), involving two carbohydrate drinks, two bolus insulin administrations, and one 45‐min exercise period. Dashed vertical black lines indicate insulin administration prior to consumption of a carbohydrate drink (1 g per kg body mass). Grey dashed box indicates the exercise session. All data are means with SEM error bars (*n* = 44). Markers of statistically significant comparisons have been omitted for clarity and detailed in subsequent text.

Participants arrived fasted at the research facility with similar baseline blood glucose concentrations across conditions (U50 9.8 ± 3.2, U75 9.5 ± 3.0, A50 10.0 ± 3.3, A75 9.4 ± 3.5 mmol L^−1^; *p* = 0.544). Figure 1 depicts the changes in blood glucose concentrations relative to rest (BG_R0_) and serum insulin concentrations relative to rest (INS_R0_) over the whole trial day (0–480 min).

### 3.4 Pre‐exercise blood glucose (0–60 min)

All trials showed a significant increase in blood glucose concentrations from baseline to 60 min post‐injection, with greater rises observed in both the U75 and A75 conditions compared to both U50 and A50 (Table 3).

**TABLE 3:** Blood glucose concentrations relative to baseline (0 min) in the first post‐prandial period (0–60 min).

Parameter	Condition				*p* value
	U50	U75	A50	A75	
Timepoint metrics					
ΔBG at 15 min (mmol L^−1^)	+1.9 ± 1.0	+2.0 ± 0.9	+2.1 ± 1.0	+2.1 ± 0.8	0.504
ΔBG at 30 min (mmol L^−1^)	+4.3 ± 1.5	+4.5 ± 1.4	+4.7 ± 1.6	+4.9 ± 1.2	0.156
ΔBG at 45 min (mmol L^−1^)	+6.1 ± 2.2	+6.5 ± 1.7	+6.3 ± 2.1	+7.0 ± 1.6	0.080
ΔBG at 60 min (mmol L^−1^)	+7.1 ± 2.8^a^	+8.3 ± 2.2^b^	+7.0 ± 2.6^bc^	+8.6 ± 1.9^ac^	<0.001*
AUC metrics					
BG_R0_ AUC_0–15min_ (mmol min L^−1^)	11 ± 7	12 ± 7	12 ± 8	12 ± 6	0.727
BG_R0_ AUC_15–30min_ (mmol min L^−1^)	48 ± 17	50 ± 16	52 ± 20	54 ± 15	0.237
BG_R0_ AUC_30–45min_ (mmol min L^−1^)	79 ± 27	85 ± 26	85 ± 27	91 ± 19	0.090
BG_R0_ AUC_45–60min_ (mmol min L^−1^)	98 ± 38^a^	111 ± 27	100 ± 33^b^	116 ± 26^ab^	0.003*
BG_R0_ AUC_0–60min_ (mmol min L^−1^)	240 ± 80	262 ± 70	251 ± 82	274 ± 57	0.088

*Note*: All data relativised to resting blood glucose concentration taken at baseline (BG_R0_). ^a,b,c^ represent statistically significant (*p* ≤ 0.05) post‐hoc comparison between two conditions.

Abbreviations: AUC, area under the curve (incremental); BG, blood glucose.

* Denotes statistical significance.

### 3.5 Blood glucose during the post‐exercise period (105–240 min)

When compared against baseline concentrations (0 min), relative blood glucose was only higher at the end of the first prandial period (240 min) in U75 and A75 conditions (U50 + 0.4 ± 3.8, U75 + 3.6 ± 5.1, A50–0.3 ± 4.8, A75 + 3.2 ± 4.7 mmol L^−1^; *p* < 0.001). AUC was also lower in both 50% dose reduction arms in the first prandial period compared to both 75% reduction arms (i.e., U50 and A50 vs. U75 and A75; all *p* ≤ 0.05) and comparisons between insulins were similar (i.e., U50 and U75 vs. A50 and A75; all *p* > 0.05).

### 3.6 Blood glucose during the second post‐prandial period (240–480 min)

At 240 min (the sample taken immediately before second prandial insulin injection and carbohydrate drink) blood glucose concentrations were different between conditions (U50 10.2 ± 4.6, U75 13.1 ± 5.8, A50 9.6 ± 4.5, A75 12.3 ± 5.3 mmol L^−1^; *p* < 0.001) with both 50% dose arms lower than both 75% reduced dose arms (all *p* ≤ 0.05). URA‐IAsp and IAsp comparisons within each equivalent dose condition remained similar (both *p* > 0.05). Inter‐trial AUC differences only occurred 60 min post‐injection in the second post‐prandial period (Table S3). BG_R0_ concentrations were lower in both 50% reduced dose arms than both 75% arms at the end of the trial day (480 min) (U50–0.3 ± 4.5, U75 + 3.5 ± 5.2, A50–0.2 ± 5.5, A75 + 3.0 ± 5.6; *p* < 0.001). Across the whole trial day, the number of hypoglycaemic events (≤3.9 mmol L^−1^) were weighted more towards the 50% trial arms (36 events) than the 75% trial arms (11 events) (Table S1).

### 3.7 Secondary outcomes: Trial day serum insulin concentrations

#### 3.7.1 Pre‐exercise serum insulin (0–60 min)

Participants displayed similar serum insulin concentrations across conditions at baseline (U50 49.2 ± 26.9, U75 45.5 ± 20.5, A50 46.7 ± 22.6, A75 46.5 ± 23.1 pmol L^−1^; *p* = 0.267). Insulin concentrations relative to baseline (INS_R0_) rose similarly in 50% reduction arms to a greater extent than 75% reduction arms (*p* ≤ 0.05 for all inter‐dose comparisons) to the start of exercise at 60 min (U50 + 21.3 ± 13.2, U75 + 13.7 ± 11.0, A50 + 20.6 ± 12.6, A75 + 11.6 ± 7.9 pmol L^−1^; *p* < 0.001). Table 4 details the insulin concentration metrics between rest (0 min) and exercise start (60 min).

**TABLE 4:** Serum insulin concentrations relative to baseline (0 min) in the first post‐prandial period (0–60 min).

Parameter	U50	U75	A50	A75	P value
Timepoint metrics					
ΔSerum insulin at 15 min (pmol L^−1^)	+13.2 ± 8.6^abc^	+8.1 ± 7.2^a^	+8.4 ± 11.4^b^	+5.8 ± 5.5^c^	<0.001*
ΔSerum insulin at 30 min (pmol L^−1^)	+21.1 ± 12.3^ab^	+14.3 ± 11.8^ac^	+19.6 ± 15.4^cd^	+9.3 ± 7.9^bd^	<0.001*
ΔSerum insulin at 45 min (pmol L^−1^)	+20.4 ± 10.7^ab^	+12.8 ± 10.5^ac^	+21.2 ± 15.6^cd^	+11.2 ± 8.0^bd^	<0.001*
ΔSerum insulin at 60 min (pmol L^−1^)	+21.3 ± 13.2^ab^	+13.7 ± 11.0^ac^	+20.6 ± 12.6^cd^	+11.6 ± 7.9^bd^	<0.001*
Pre‐exercise *C*_max_ metrics					
*t*_max *C*max0–60min_ (min)	42.1 ± 13.4	42.9 ± 14.7	44.2 ± 12.6	43.6 ± 11.6	0.889
*C*_max0–60min_ (pmol L^−1^)	+26.6 ± 12.5^ab^	+18.2 ± 11.8^ac^	+26.7 ± 16.0^cd^	+14.8 ± 7.9^bd^	<0.001*
AUC metrics					
INS_R0_ AUC_0–15min_ (pmol min L^−1^)	92 ± 74^abc^	62 ± 58^ad^	40 ± 66^b^	24 ± 40^cd^	<0.001*
INS_R0_ AUC_15–30min_ (pmol min L^−1^)	261 ± 156^ab^	171 ± 130^ac^	203 ± 195^d^	118 ± 84.8^bcd^	<0.001*
INS_R0_ AUC_30–45min_ (pmol min L^−1^)	311 ± 177^ab^	197 ± 145^ac^	309 ± 232^cd^	162 ± 108^bd^	<0.001*
INS_R0_ AUC_45–60min_ (pmol min L^−1^)	310 ± 187^ab^	199 ± 158^ac^	315 ± 200^cd^	172 ± 114^bd^	<0.001*
INS_R0_ AUC_0–60min_ (pmol min L^−1^)	992 ± 580^ab^	628 ± 475^a^	877 ± 703^c^	471 ± 325^bc^	<0.001*

*Note*: All data relativised to insulin concentrations at baseline (INS_R0_). *t*_max *C*max60min_, time until maximum concentration before start of exercise (60 min); *C*_max60min_, maximum concentration before start of exercise (60 min). ^a,b,c,d^ represent statistically significant (*p* ≤ 0.05) post‐hoc comparison between two conditions.

Abbreviation: AUC, area under the curve.

* Denotes statistical significance for main effect.

#### 3.7.2 Insulin concentrations during exercise (60–105 min)

From exercise start to end, serum insulin concentrations relativised to baseline (INS_R0_) increased from 62.5 to 67.1 pmol L^−1^ averaged across conditions (*p* < 0.001); the increase was similar across all conditions (U50 + 3.4 ± 9.1, U75 + 6.9 ± 15.1, A50 + 3.4 ± 8.8, A75 + 5.2 ± 7.7 pmol L^−1^; *p* = 0.328). There were no differences in incremental AUC from exercise start to end (U50 + 85 ± 239, U75 + 187 ± 396, A50 + 112 ± 260, A75 + 132 ± 188 pmol min L^−1^; *p* = 0.410).

#### 3.7.3 Insulin concentrations post‐exercise

AUC during the first prandial period (0–240 min) was higher in the 50% dose reduction arms compared to the 75% reduction arms, without an insulin type effect (U50 519 ± 234, U75 679 ± 179, A50 534 ± 237, A75 671 ± 189 pmol min L^−1^; *p* < 0.001). At 240 min (the sample taken immediately before second prandial injection and drink meal), serum insulin, relativised to baseline (0 min), was similar between trial arms (U50 + 16.0 ± +17.8, U75 + 16.5 ± 24.3, A50 + 12.5 ± 9.7, A75 + 14.6 ± 13.3 pmol L^−1^; *p* = 0.518). Similarly to the first prandial period, incremental AUC in the second prandial period (relativised to concentrations at 240 min) was higher in the 50% dose conditions after 60 min post‐injection and 105 min post‐injection (both *p* < 0.001).

## 4 DISCUSSION

This study is the first to compare an URA‐IAsp and a IAsp in different dose reductions around exercise in individuals with T1D using MDI. Our findings demonstrated that mealtime dose reduction of either 50% or 75% ahead of exercise has a greater effect on blood glucose concentrations within and around exercise than insulin type. Furthermore, although early post‐prandial serum insulin concentrations were influenced by insulin type, these differences diminished in exercise and later post‐prandial periods.

### 4.1 Pre‐exercise

Trials using URA‐IAsp (U50 and U75) exhibited greater serum insulin AUC from rest to 15 min post‐injection than equivalent doses in IAsp (A50 and A75, respectively). By 30 min, point concentrations between insulin types were similar across conditions and, subsequently, at 45 and 60 min, insulin concentrations expressed as relative point concentrations and AUC were similar between URA‐IAsp and IAsp when administered at the same dose. Greater early exposure has been well demonstrated in URA‐IAsp versus IAsp up to 2 h post‐injection (e.g., Heise et al.^17^); however, dosing quantity may influence the duration of AUC differences between the two insulins.^18^ A euglycaemic clamp study demonstrated that injections of 0.1, 0.2, or 0.4 UI per kilogram of body mass produced higher insulin exposure up to 30 min post‐injection in URA‐IAsp compared to IAsp, yet, by 60 min, there were no differences between the two insulin types under the 0.1 UI kg^−1^ condition, despite ongoing differentiation in the 0.2 and 0.4 UI kg^−1^ arms.^19^ The similarity in AUC after 60 min between insulins aligns with our data, which is closest to the 0.1 UI kg^−1^ trial arm, albeit still some magnitudes less in dosage.

Despite differences in early insulin exposure in the pre‐exercise period, differences in blood glucose concentrations between trials were not significant until 60 min after injection. These data might be explained by a low glucose‐lowering capability of reduced doses of both insulins relative to the rapid influx of a large amount of carbohydrate as glucose into the circulation from the digestive system. Furthermore, the carbohydrate‐heavy meal (1 g kg^−1^ carbohydrates [~90%], ~0.1 g kg^−1^ protein [~10%], 0 g kg^−1^ fat [0%]) contained predominantly high‐glycaemic index ingredients (i.e., glucose syrup and maltodextrin) which may have further contributed to the steep rise in blood glucose concentrations over a 1‐h rested period.

### 4.2 Impact of insulin type and dose on exercise glycaemia

This is the first study to compare the glycaemic effects of different reduced dosages of URA‐IAsp and IAsp around exercise using MDI regimen. Moderate‐intensity cycling at around 60% V̇O_2peak_ induced a reduction in blood glucose in all conditions. Regarding the comparison between conditions, blood glucose concentrations fell similarly between insulin types in both −50% and −75% conditions. Post‐hoc testing revealed that, while blood glucose declined similarly in U50 compared to all other conditions, A50 fell to a greater extent than both U75 and A75 conditions. While there was a trend for A50 (−5.1 ± 3.0 mmol L^−1^) blood glucose to decline further than U50 (−4.0 ± 2.8 mmol L^−1^), this did not reach statistical significance (*p* = 0.067). Molveau et al.^9^ recently performed a comparable study, where 50% reduced URA‐IAsp or IAsp was injected 60 or 120 min prior to 60 min of moderate‐intensity continuous exercise on a cycle ergometer. When combining the timing effects, URA‐IAsp was reported to have declined to a lesser extent compared to IAsp (−4.1 ± 2.3 vs. −4.4 ± 2.8 mmol L^−1^; *p* = 0.037), a result that is in partial agreement with our findings.

IAsp is recommended to be taken up to 15 min prior to the start of mealtimes.^20^ Conversely, URA‐IAsp may be taken at the start of mealtimes and still provide a glucose‐lowering effect that effectively matches glucose absorption profiles.^21^ The potential for a shorter time between ultra‐rapid insulin injection and a bout of exercise may be advantageous to individuals adjusting insulin dosing at short notice. Our data suggest that even when taken immediately prior to a drink meal, URA‐IAsp and IAsp produce similar glucose‐lowering effects in reduced doses, potentially providing some assurance for the individual exercising in the post‐prandial period who is switching from a rapid‐acting insulin to an ultra‐rapid‐acting insulin.

Serum insulin concentrations rose similarly (by approximately 7%) during cycling in all conditions—even with heavy insulin dose reductions—with comparable AUC during exercise between URA‐IAsp and IAsp conditions. The exercise sessions in this study took place between 60 and 105 min after injection. In PD/PK studies performed at rest, this would typically be a period where rapid‐acting insulin concentrations would peak, plateau, and subsequently begin to decline. Furthermore, in people without T1D performing moderate‐intensity exercise, pancreatic insulin output decreases to prevent an unregulated decline in blood glucose concentrations. The phenomenon of a transient rise in insulinaemia demonstrated during the exercise period in our study is congruent with findings from other studies^22^ and is potentially attributable to a combination of exercise‐induced relative hypovolaemia (i.e., the lowering of blood volume typical during acute exercise will increase the concentration of blood insulin, without more insulin entering the system) and increased rate of insulin absorption from the injection depot.^23^, ^24^

### 4.3 Post‐exercise

Results from this study suggest that a 50% reduction in URA‐IAsp will result in post‐exercise glucose concentrations at 2 h 15 min (135 min) to be similar to those at baseline, that is, before the pre‐exercise meal (~Δ +0.4 mmol L^−1^). At the same timepoint, blood glucose concentrations were elevated under the URA‐IAsp 75% reduction condition (Δ +3.6 mmol L^−1^; *p* < 0.001). Molveau et al.^9^ observed concentrations most similar to baseline after 90 min post‐exercise when injecting a 50% reduction in URA‐IAsp dose 60 min pre‐exercise (Δ −2.8 mmol L^−1^) compared to 120 min (−4.6 mmol L^−1^; *p* = 0.001). Combined, these data indicate that a person taking a 50% reduced dose of URA‐IAsp 60 min prior to a bout of moderate‐intensity continuous exercise can attain glucose concentrations similar to baseline ~2 h (between 90 and 135 min) after exercise.

### 4.4 Second prandial period

Inter‐individual variability in AUC metrics was significant throughout the trial days in both glucose and insulin concentrations, emphasising the need for personalised insulin adjustments around exercise. Nevertheless, the pattern of elevated serum insulin AUC in 50% over 75% reduction condition continued consistently throughout the second prandial period. The similarity between URA‐IAsp and IAsp at the point of the second injection allows previously established duration of insulin action guidelines to be applied to the reduced doses of URA‐IAsp as with IAsp, where the individual with T1D should be mindful that residual insulin activity may be present 4 h after the first bolus injection of the day—particularly in pump users—to avoid insulin stacking.^25^, ^26^

### 4.5 Strengths and limitations

This study has several strengths. This is the first laboratory‐controlled exercise‐based study to compare two insulin dose reductions in cross‐over comparison with an ultra‐rapid‐acting insulin, URA‐IAsp, and a rapid‐acting insulin, IAsp. A high venous sampling frequency was used, up to every 5 min, to gain high resolution of blood glucose and serum insulin changes throughout periods where pharmacokinetic differences between URA‐IAsp and IAsp have been shown to be marginal. However, the study is not without limitations. The use of a high glycaemic index drink with a high carbohydrate load at mealtimes frequently led to level 2 hyperglycaemia (>13.9 mmol L^−1^) throughout the trial day.^27^ Nevertheless, this study provides a platform to examine the glucose lowering effects of the different insulin conditions under a nutritionally controlled environment.

We included both males and females in this study which provides improved applicability than the inclusion of any single sex alone; however, we did not account for female menstrual cycle, which may impact blood glucose concentrations during the day and during exercise.^28^ Lastly, data collection was halted during government‐enforced lockdown due to COVID‐19, significantly protracting the data collection period over which the study was performed.

## 5 CONCLUSION

This is the first study to compare the use of URA‐IAsp and IAsp when using different bolus insulin dose reductions around exercise. Altering mealtime insulin dose reductions between 50% and 75% exerts a greater influence on glycaemia around post‐prandial exercise than altering between ultra‐ or rapid‐acting insulins, and that insulin dose reductions around acute moderate‐intensity exercise can be used with similar glucose‐lowering effects in URA‐IAsp and IAsp.

## FUNDING INFORMATION

This study was funded by Novo Nordisk A/S as an Investigator Sponsored Study.

## CONFLICT OF INTEREST STATEMENT

Othmar Moser: Clinical trial support: Sêr Cymru II COFUND Fellowship/European Union, Novo Nordisk A/S, Novo Nordisk AT, Abbott Diabetes Care, Sanofi, Dexcom, Team Novo Nordisk, SAIL, Maisels Brauerei, Medtronic AT, EFSD/EASD, Falke, BISp, perfood, Ypsomed, Sinocare, Roche. Presenters' honoraria: Medtronic AT, Medtronic Int., Eli Lilly, Novo Nordisk, Sanofi, TAD Pharma, ADA, Diatec, Berufsverband deutscher Internist*innen, Dexcom, AstraZeneca, Ypsomed, Insulet, Diabetologen Hessen, Abbott, Hanako. Conference travel support: Novo Nordisk A/S, Novo Nordisk AT, Novo Nordisk UK, Medtronic AT, Sanofi, EASD, OEDG, DDG. Advisory board: Sanofi, TAD Pharma, Dexcom, perfood, Medtronic, Hedia, Roche. Harald Sourij receives research support (to the Medical University of Graz) from Boehringer Ingelheim, Eli Lilly, Novo Nordisk and Sanofi and honoraria from AstraZeneca, Amarin, Bayer, Boehringer Ingelheim, Daiichy Sankyo, Eli Lilly, Novartis, Novo Nordisk, and Sanofi. Stephen C. Bain has received investigator fees for Novo Nordisk clinical trials (to institution) as well as personal fees for speaking and attendance at Advisory Boards.

## Supporting information


**Table S1.** Carbohydrate treatment for the occurrence of hypoglycaemia (blood glucose ≤3.9 mmol L^−1^).
**Table S2.** Spirometry and exertion data during exercise.
**Table S3.** Blood glucose concentrations for the second post‐prandial period (240–480 min).
**Figure S1.** Trial day protocol schematic.

## Data Availability

The data that support the findings of this study are available from the corresponding author upon reasonable request.

## References

[1] Haahr H , Heise T . Fast‐acting insulin aspart: a review of its pharmacokinetic and pharmacodynamic properties and the clinical consequences. Clin Pharmacokinet. 2020;59(2):155‐172. doi:10.1007/s40262-019-00834-5 31667789 PMC7007438

[2] Heise T , Linnebjerg H , Coutant D , et al. Ultra rapid lispro lowers postprandial glucose and more closely matches normal physiological glucose response compared to other rapid insulin analogues: a phase 1 randomized, crossover study. Diabetes Obes Metab. 2020;22(10):1789‐1798. doi:10.1111/dom.14094 32436641 PMC7540588

[3] Heise T , Hövelmann U , Zijlstra E , Stender‐Petersen K , Jacobsen JB , Haahr H . A comparison of pharmacokinetic and pharmacodynamic properties between faster‐acting insulin aspart and insulin aspart in elderly subjects with type 1 diabetes mellitus. Drugs Aging. 2017;34(1):29‐38. doi:10.1007/s40266-016-0418-6 27873152 PMC5222895

[4] Russell‐Jones D , Bode BW , De Block C , et al. Fast‐acting insulin aspart improves glycemic control in basal‐bolus treatment for type 1 diabetes: results of a 26‐week multicenter, active‐controlled, treat‐to‐target, randomized, parallel‐group trial (onset 1). Diabetes Care. 2017;40(7):943‐950. doi:10.2337/dc16-1771 28356319

[5] Mathieu C , Bode BW , Franek E , et al. Efficacy and safety of fast‐acting insulin aspart in comparison with insulin aspart in type 1 diabetes (onset 1): a 52‐week, randomized, treat‐to‐target, phase III trial. Diabetes Obes Metab. 2018;20:1148‐1155. doi:10.1111/dom.13205 29316130 PMC5947306

[6] Buse JB , Carlson AL , Komatsu M , et al. Fast‐acting insulin aspart versus insulin aspart in the setting of insulin degludec‐treated type 1 diabetes: efficacy and safety from a randomized double‐blind trial. Diabetes Obes Metab. 2018;20:2885‐2893. doi:10.1111/dom.13545 30259644 PMC6231963

[7] Riddell MC , Gallen IW , Smart CE , et al. Exercise management in type 1 diabetes: a consensus statement. Lancet Diabetes Endocrinol. 2017;5(5):377‐390. doi:10.1016/S2213-8587(17)30014-1 28126459

[8] Colberg SR , Sigal RJ , Yardley JE , et al. Physical activity/exercise and diabetes: a position statement of the American Diabetes Association. Diabetes Care. 2016;39(11):2065‐2079. doi:10.2337/dc16-1728 27926890 PMC6908414

[9] Molveau J , Myette‐Côté É , Tagougui S , et al. Assessing the influence of insulin type (ultra‐rapid vs rapid insulin) and exercise timing on postprandial exercise‐induced hypoglycaemia risk in individuals with type 1 diabetes: a randomised controlled trial. Diabetologia. 2024;67:2408‐2419. doi:10.1007/s00125-024-06234-0 39069599

[10] Hofmann P , Tschakert G . Special needs to prescribe exercise intensity for scientific studies. Cardiol Res Pract. 2011;2011:1‐10. doi:10.4061/2011/209302 PMC3010619 21197479

[11] Borg GAV . Psychophysical bases of perceived exertion. Plast Reconstr Surg. 1982;14(5):377‐381. 7154893

[12] Yazici B , Yolacan S . A comparison of various tests of normality. J Stat Comput Simul. 2007;77(2):175‐183. doi:10.1080/10629360600678310

[13] Allison D , Paultre F , Maggio C , Mezzitis N , Pi‐Sunyer F . The use of areas under curves in diabetes research. Diabetes Care. 1995;18(2):245‐250. 7729306 10.2337/diacare.18.2.245

[14] Hampel FR . The influence curve and its role in robust estimation. J Am Stat Assoc. 1974;69(346):383‐393. doi:10.1080/01621459.1974.10482962

[15] Nurhaliza RA , Octava MQH , Hilmy FM , Farooq U , Alfian G . Application of the outlier detection method for web‐based blood glucose level monitoring system. Bull Electr Eng Inform. 2024;13(4):2809‐2816. doi:10.11591/eei.v13i4.7717

[16] Jadhav MM , Sarwade AN , Sardar VM , Jadhav HM . Development of a non‐invasive blood glucose monitoring device using machine learning technology. Passer J Basic Appl Sci. 2024;6(1):58‐73. doi:10.24271/PSR.2023.388351.1272

[17] Heise T , Pieber TR , Danne T , Erichsen L , Haahr H . A pooled analysis of clinical pharmacology trials investigating the pharmacokinetic and pharmacodynamic characteristics of fast‐acting insulin aspart in adults with type 1 diabetes. Clin Pharmacokinet. 2017;56(5):551‐559. doi:10.1007/s40262-017-0514-8 28205039 PMC5385193

[18] Heise T , Hövelmann U , Brøndsted L , Adrian CL , Nosek L , Haahr H . Faster‐acting insulin aspart: earlier onset of appearance and greater early pharmacokinetic and pharmacodynamic effects than insulin aspart. Diabetes Obes Metab. 2015;17(7):682‐688. doi:10.1111/dom.12468 25846340 PMC5054830

[19] Heise T , Stender‐Petersen K , Hövelmann U , et al. Pharmacokinetic and pharmacodynamic properties of faster‐acting insulin aspart versus insulin aspart across a clinically relevant dose range in subjects with type 1 diabetes mellitus. Clin Pharmacokinet. 2017;56(6):649‐660. doi:10.1007/s40262-016-0473-5 27878566 PMC5425492

[20] NHS . How and when to take rapid‐acting insulin. 2023. Accessed July 6, 2023. https://www.nhs.uk/medicines/insulin/rapid-acting-insulin/how-and-when-to-take-rapid-acting-insulin/

[21] Novomedlink . Fiasp: insulin aspart injection 100units/mL. Accessed October 30, 2021. https://www.novomedlink.com/diabetes/products/treatments/fiasp.html

[22] Riddell MC , Scott SN , Fournier PA , et al. The competitive athlete with type 1 diabetes. Diabetologia. 2020;63:1475‐1490. 32533229 10.1007/s00125-020-05183-8 PMC7351823

[23] Lindinger MI . A century of exercise physiology: key concepts in muscle cell volume regulation. Eur J Appl Physiol. 2022;122(3):541‐559. doi:10.1007/s00421-021-04863-6 35037123

[24] Pitt JP , McCarthy OM , Hoeg‐Jensen T , Wellman BM , Bracken RM . Factors influencing insulin absorption around exercise in type 1 diabetes. Front Endocrinol (Lausanne). 2020;11:793‐802. doi:10.3389/fendo.2020.573275 PMC7609903 33193089

[25] Walsh J , Roberts R , Heinemann L . Confusion regarding duration of insulin action: a potential source for major insulin dose errors by bolus calculators. J Diabetes Sci Technol. 2014;8(1):170‐178. doi:10.1177/1932296813514319 24876553 PMC4454113

[26] Walsh J , Roberts R , Bailey TS . Insulin titration guidelines for patients with type 1 diabetes: it is about time ! J Diabetes Sci Technol. 2023;17(4):1066‐1076. doi:10.1177/19322968221087261 35369773 PMC10348003

[27] Battelino T , Danne T , Bergenstal RM , et al. Clinical targets for continuous glucose monitoring data interpretation: recommendations from the international consensus on time in range. Diabetes Care. 2019;42(8):1593‐1601. doi:10.2337/dci19-0028 31177185 PMC6973648

[28] Toor S , Yardley JE . Type 1 diabetes and the menstrual cycle: where/how does exercise fit in? Int J Environ Res Public Health. 2023;20:2772‐2784. 36833469 10.3390/ijerph20042772 PMC9957258

