# Supplementary material for: Comparison of pharmacodynamics and pharmacokinetics of ultra‐rapid‐acting insulin aspart and rapid‐acting insulin aspart around continuous moderate intensity exercise in adults with type 1 diabetes: A randomised controlled trial
Source: Diabetes Obes Metab. 2026 Feb 10;28(4):3002–10. doi: 10.1111/dom.70487 (PMC12992166; doi:10.1111/dom.70487)
Supplement: Supplementary file 1 — Table S1. Carbohydrate treatment for the occurrence of hypoglycaemia (blood glucose ≤3.9 mmol L−1). Table S2. Spirometry and exertion data during exercise. Table S3. Blood glucose concentrations for the second post‐prandial period (240–480 min). Figure S1. Trial day protocol schematic. [file DOM-28-3002-s001.doc]

Supplementary Material for: Comparison of pharmacodynamics and pharmacokinetics of ultra-rapid-acting insulin aspart and rapid-acting insulin aspart around continuous moderate intensity exercise in adults with type 1 diabetes: a randomised controlled trial – Jason Pitt – October 2025

**Supplementary Table 1**: Carbohydrate treatment for the occurrence of hypoglycaemia (blood glucose ≤3.9 mmol.L^-1^).

| **Parameter** | **F50** | **F75** | **A50** | **A75** |
| --- | --- | --- | --- | --- |
| **Number of hypoglycaemic episodes treated for hypoglycaemia** | 16 | 7 | 20 | 4 |
| **Number of participants experiencing hypoglycaemia** | 8 | 3 | 8 | 4 |

**Supplementary Table 2**: Spirometry and exertion data during exercise.

| **Parameter** | **F50** | **F75** | **A50** | **A75** | **P value** |
| --- | --- | --- | --- | --- | --- |
| **V̇O_2_ (L.min^-1^)** | 1.74 ± 0.39 | 1.72 ± 0.36 | 1.73 ± 0.38 | 1.73 ± 0.38 | 0.688 |
| **V̇CO_2_ (L.min^-1^)** | 1.65 ± 0.37 | 1.62 ± 0.36 | 1.65 ± 0.36 | 1.63 ± 0.37 | 0.534 |
| **V̇O_2_ (mL.kg^-1^.min^-1^)** | 23.3 ± 4.7 | 22.9 ± 4.4 | 23.0 ± 4.3 | 23.0 ± 4.4 | 0.570 |
| **%V̇O_2peak_** | 61.9 ± 6.1 | 61.2 ± 7.3 | 61.4 ± 6.6 | 61.4 ± 7.0 | 0.763 |
| **RER** | 0.94 ± 0.04 | 0.94 ± 0.04 | 0.95 ± 0.04 | 0.94 ± 0.04 | 0.364 |
| **V̇E (L.min^-1^)** | 49.9 ± 9.7 | 49.2 ± 9.5 | 50.4 ± 10.2 | 49.4 ± 9.5 | 0.410 |
| **Energy expenditure (kcals.min^-1^)** | 8.6 ± 1.9 | 8.6 ± 1.9 | 8.7 ± 1.9 | 8.6 ± 1.9 | 0.788 |
| **Energy expenditure (kJ.min^-1^)** | 36.2 ± 8.1 | 35.8 ± 7.9 | 36.2 ± 7.9 | 36.0 ± 8.0 | 0.787 |
| **Carbohydrate oxidation (g.min^-1^)** | 1.76 ± 0.47 | 1.74 ± 0.49 | 1.81 ± 0.47 | 1.76 ± 0.51 | 0.385 |
| **Lipid oxidation (g.min^-1^)** | 0.16 ± 0.12 | 0.16 ± 0.10 | 0.14 ± 0.11 | 0.16 ± 0.12 | 0.441 |
| **Carbohydrate oxidation percentage energy expenditure (%)** | 82.9 ± 12.5 | 82.4 ± 11.3 | 85.0 ± 11.3 | 82.8 ± 13.6 | 0.348 |
| **Lipid oxidation percentage energy expenditure (%)** | 17.1 ± 12.5 | 17.6 ± 11.3 | 15.0 ± 11.3 | 17.2 ± 13.6 | 0.348 |
| **Blood lactate during exercise (mmol.L^-1^)** | 2.9 ± 1.0 | 2.8 ± 1.1 | 2.7 ± 0.9 | 2.7 ± 1.0 | 0.331 |
| **RPE during exercise** | 12.4 ± 1.8 | 12.3 ± 2.1 | 12.2 ± 2.1 | 12.1 ± 2.0 | 0.173 |
| **HR (beats.min^-1^)** | 136 ± 16 | 134 ± 16 | 136 ± 17 | 132 ± 23 | 0.294 |
| **O_2_pulse (mL.beat^-1^)** | 13.0 ± 3.3 | 12.8 ± 2.9 | 12.8 ± 3.2 | 13.3 ± 4.1 | 0.446 |
| **Cycling cadence (revolutions.min^-1^)** | 72.4 ± 4.1 | 72.8 ± 4.1 | 73.0 ± 4.0 | 72.6 ± 3.4 | 0.638 |
| **Power during exercise (W)** | 113 ± 34 | 113 ± 34 | 113 ± 34 | 113 ± 34 | - |

*Data are presented as Mean±SD. * Denotes statistical significance. HR, heart rate; RER, Respiratory exchange ratio; RPE, Rating of Perceived Exertion; V̇CO_2_, (rate of) volume of carbon dioxide output; V̇E, minute ventilation; V̇O_2_, (rate of) volume of oxygen uptake. Data for one participant were removed after the point of carbohydrate ingestion (i.e., 35 min after the start of exercise) to avoid hypoglycaemia.*

**Supplementary Table 3:** Blood glucose concentrations for the second post-prandial period (240-480 min).

| **Parameter** | **F50** | **F75** | **A50** | **A75** | **P value** |
| --- | --- | --- | --- | --- | --- |
| **t _max Cmax240-345min_ (min)** | 89.0 ± 18.3 | 94.2 ± 10.6 | 87.9 ± 18.4 | 93.3 ± 14.8 | 0.113 |
| **C_max240-345min_ (mmol.L^-1^)** | +8.1 ± 2.8^ab^ | +9.5 ± 2.6^a^ | +9.0 ± 2.8 | +9.8 ± 2.7^b^ | **0.004*** |
| **AUC_240-255min_ (mmol.min.L^-1^)** | 2.8 ± 7.2 | 3.2 ± 6.7 | 6.0 ± 6.8 | 5.4 ± 7.9 | 0.146 |
| **AUC_240-270min_ (mmol.min.L^-1^)** | 30.5 ± 30.0 | 36.5 ± 24.5 | 45.7 ± 25.7 | 42.6 ± 28.9 | 0.056 |
| **AUC_240-300min_ (mmol.min.L^-1^)** | 162 ± 100^a^ | 204 ± 68 | 213 ± 83 | 226 ± 85^a^ | **0.006*** |
| **AUC_240-345min_ (mmol.min.L^-1^)** | 438 ± 207^ab^ | 567 ± 193^a^ | 538 ± 185 | 609 ± 185^b^ | **0.006*** |
| **ΔBG from start of second prandial (240 min) to end of visit (480min) (mmol.L^-1^)** | -0.5 ± 2.7 | +0.5 ± 2.8 | +0.3 ± 2.5 | +0.3 ± 3.0 | 0.266 |
| **BG_R0_ at end of visit ([480 min] mmol.L^-1^)** | -0.3 ± 4.5^ab^ | 3.5 ± 5.2^ac^ | -0.2 ± 5.5^cd^ | 3.0 ± 5.6^bd^ | **<0.001*** |
| **Non-relativised BG at end of visit ([480 min] mmol.L^-1^)** | 9.4 ± 4.5^ab^ | 12.6 ± 5.1^ac^ | 9.6 ± 4.5^cd^ | 11.9 ± 5.0^bd^ | **<0.001*** |

*All metrics are relativised to the start of the second prandial period (240 min), unless otherwise indicated. t_max Cmax240-345min_, individualised time until maximum blood glucose concentration between start of second prandial period (240 min) and the time equivalent to end of exercise (345 min); C_max240-345min_, individualised maximum blood glucose concentration between the start of second prandial period (240 min) and the time equivalent to end of exercise (345 min); AUC, area under the curve (incremental). * Denotes statistical significance for main effect. ^a,b,c,d^ represent statistically significant (p≤0.05) post-hoc comparison between two conditions.*


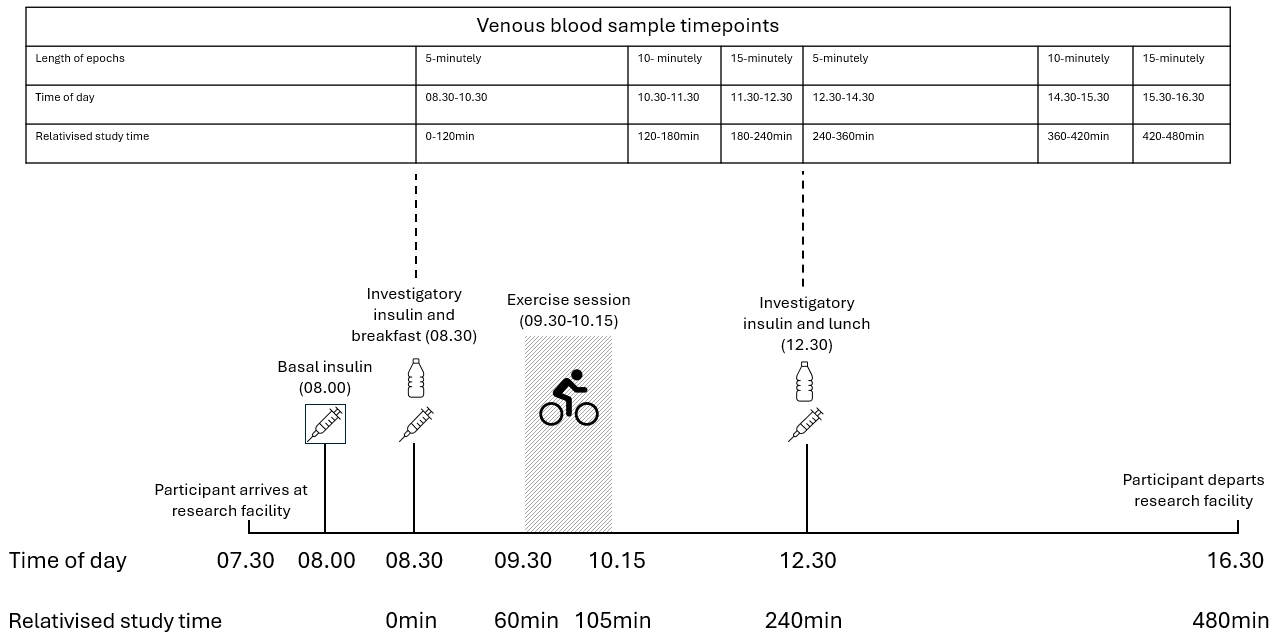
***Supplementary Figure 1: Trial day protocol schematic***
